# The impact of immune checkpoint inhibitors on prognosis in unresectable hepatocellular carcinoma treated with TACE and lenvatinib: a meta-analysis

**DOI:** 10.3389/fimmu.2025.1573505

**Published:** 2025-05-21

**Authors:** Wei Zhang, Zirong Liu, Hongjin Liu, Zhangkan Huang, Xiaozhun Huang, Lin Xu, Xu Che, Zhengyin Zhan

**Affiliations:** ^1^ Department of Hepatobiliary and Pancreatic Surgery, National Cancer Center/National Clinical Research Center for Cancer/Cancer Hospital & Shenzhen Hospital, Chinese Academy of Medical Sciences and Peking Union Medical College, Shenzhen, Guangdong, China; ^2^ Department of Medical Oncology, National Cancer Center/National Clinical Research Center for Cancer/Cancer Hospital & Shenzhen Hospital, Chinese Academy of Medical Sciences and Peking Union Medical College, Shenzhen, Guangdong, China

**Keywords:** hepatocellular carcinoma, transarterial chemoembolization, lenvatinib, immune checkpoint inhibitor, PD-1 inhibitor

## Abstract

**Background:**

Combination of multiple therapies is a common approach to treating patients with unresectable hepatocellular carcinoma (uHCC). The impact of immune checkpoint inhibitors (ICIs) on prognosis in uHCC patients treated with transarterial chemoembolization (TACE) and lenvatinib remains unclear.

**Aim:**

The purpose of this study was to compare the efficacy and safety of TACE plus lenvatinib plus ICIs (TACE+L+I) with TACE plus lenvatinib (TACE+L) in the treatment of patients with uHCC.

**Methods:**

Publicly available studies comparing the efficacy and safety of TACE+L+I and TACE+L in the treatment of uHCC were collected from the databases PubMed, Embase and Cochrane Library, with a cut-off date of November 1, 2024. Stata SE 15 software was used for analysis.

**Results:**

Fifteen studies with a total of 1365 patients were included, 688 in the TACE+L+I group and 677 in the TACE+L group. Meta-analysis showed that the TACE+L+I group was significantly higher than the TACE+L group in complete response (RR = 2.34, 95%CI:1.53, 3.59, *p* < 0.0001), partial response (RR = 1.45, 95%CI:1.28, 1.64, *p* < 0.0001), objective response rate (RR = 1.55, 95%CI:1.39, 1.73, *p* < 0.00001), and disease control rate (RR = 1.22, 95%CI:1.10, 1.36, *p* = 0.0003). The TACE+L+I group was significantly lower than the TACE+L group in progression of disease (RR = 0.39, 95%CI:0.30, 0.51, *p* < 0.00001). Moreover, TACE+L+I group was not significantly different from TACE+L group in stable disease (RR = 0.85, 95%CI:0.69, 1.03, *p* = 0.10). The TACE+L+I group was significantly higher than the TACE+L group in overall survival (HR = 2.32, 95%CI:1.95, 3.15, *p*<0.05) and progression-free survival (HR = 2.30, 95%CI:1.80, 2.93, *p*<0.05). The TACE+L+I group had a significantly higher incidence of hypothyroidism compared to the TACE+L group (RR = 1.81, 95%CI:1.20, 2.71, *p*<0.05), but there was no significant difference in other adverse events, such as hypertension, diarrhea, hand-foot syndrome, fatigue, elevated AST, elevated ALT, decreased appetite, hypothyroidism, abdominal pain, thrombocytopenia, rash, and nausea.

**Conclusion:**

ICIs significantly improved the survival outcome of uHCC treated with TACE+L, and increased the incidence of hypothyroidism. However, this conclusion still needs further validation in the future with more high-quality randomized controlled trials and longer follow-up.

## 1 Introduction

Hepatocellular carcinoma (HCC), one of the most common fatal malignancies, accounts for 75-85% of primary liver malignant tumors (1). Although surgical resection, ablation, and liver transplantation are effective on early-stage HCC, most patients with HCC are diagnosed with advanced disease and have a poor prognosis, with an expected median survival of 6–8 months (2–4). Transarterial chemoembolization (TACE) is recommended by various guidelines for the treatment of unresectable HCC (uHCC) (2–4). The use of TACE can effectively slow down the local progression of intrahepatic tumors, but may not be as effective in treating extrahepatic metastases, making it a less satisfactory treatment option (5). Hypoxia occurs in liver after TACE, which induce tumor angiogenesis and potentially lead to tumor recurrence and progression. The combination of anti-angiogenic drugs with TACE can effectively counteract the angiogenesis caused by hypoxia after TACE, resulting in better inhibition of HCC (6, 7). Lenvatinib is a new tyrosine kinase inhibitor approved in 2018 as a first-line treatment for uHCC (8). Some studies have shown that the combination of TACE and lenvatinib can induce a satisfactory effect in the treatment of uHCC (9–13), and the combination of TACE and antiangiogenic drugs has become a promising choice for the treatment of advanced-stage HCC.

Immune checkpoint inhibitors (ICIs), including programmed death 1 (PD-1) and programmed death ligand 1 (PD-L1) inhibitors, have recently shown clinical benefit in patients with a variety of solid tumors (14). Some encouraging results suggest that the combination of TACE, lenvatinib, and ICIs has promising therapeutic potential for patients with HCC (15, 16). In theory, hypoxia following TACE promotes angiogenesis and disrupts antitumor immunity. However, lenvatinib not only inhibits angiogenesis but also normalizes vasculature and reduces the immunosuppressive environment of tumors, creating a favorable setting for T cell trafficking into tumors, thereby enabling the efficacy of ICIs (17–19); thus the combination of TACE, lenvatinib, and ICIs may induce a synergistic antitumor effect on HCC, improving clinical outcomes and inducing manageable side effects. Some studies comparing the efficacy and safety of TACE+L+I versus TACE combined with lenvatinib (TACE+L) in the treatment of patients with uHCC are available, but with inconsistent conclusions. Therefore, the purpose of this meta-analysis was to evaluate the efficacy and safety of ICIs in patients with uHCC treated with TACE+L, to be used as a clinical reference.

## 2 Materials and methods

A systematic evaluation and meta-analysis of preferred reporting items was performed according to the PRISMA guidelines (20). This study did not require formal institutional review board approval or patient informed consent because it was a secondary study using publicly available data.

### 2.1 Search strategy

A literature search on the EMBASE, PubMed, and Cochrane Library databases was performed to identify relevant available articles up to November 1, 2024. The search strategy for each database is shown in the **Supplementary File S1**. The authors were contacted to obtain extra information if necessary. If multiple studies were performed by the same authors or medical centers with duplicates in patients, the highest quality study was selected.

### 2.2 Inclusion criteria

(1) Study population: confirmed diagnosis of uHCC; (2) Publicly available literature reporting comparative efficacy of TACE+L+I and TACE+L; (3) No restriction on the study sample size; (4) No restriction on the duration of the follow-up; (5) No restriction on the type of language used to write the articles; (6) Human studies only; and (7) Study results were evaluated by the Response to Criteria for Evaluation of the Efficacy of Solid Tumors (RECIST), Common Terminology Criteria for Adverse Events v5.0.

### 2.3 Extraction criteria

(1) Studies with incomplete information, no access to valid data, no response from the authors after contacting them, duplicates and unpublished studies; (2) single-arm studies of TACE+L+I or TACE+L; (3) other treatments such as radiofrequency ablation; and (4) reviews, case reports, and animal experiments.

### 2.4 Quality assessment

In all the included studies, RCTs conducted a risk assessment of the risk according to the “risk assessment tool” recommended by the Cochrane Collaboration Network. The Cohort Studies are based on the Newcastle-Ottawa Scale. The results are shown in the **Supplementary File S2**.

### 2.5 Statistical analysis

Statistical analysis in this meta-analysis was performed using Stata SE 15 software. Relative risk (RR) was calculated in comparison of dichotomous variables by Mantel-Haenszel method, and hazard ratio (HR) was calculated in the comparison of survival variables by Inverse Variance method. The level of heterogeneity among studies was evaluated using *I* ^2^ statistics. A randomized model was used in this study. Sensitivity analysis was performed by removing 1 study at a time to assess whether the results were markedly affected by a single study. Funnel plots were used to qualitatively assess publication bias, and the results are shown in the **Supplementary File S3**. Begg’s test and Egger’s test were quantitatively used to assess publication bias in the included studies, and their significance level was limited to 0.05, as shown in the **Supplementary File S4**.

## 3 Results

### 3.1 Search results and study selection

**Figure 1:**
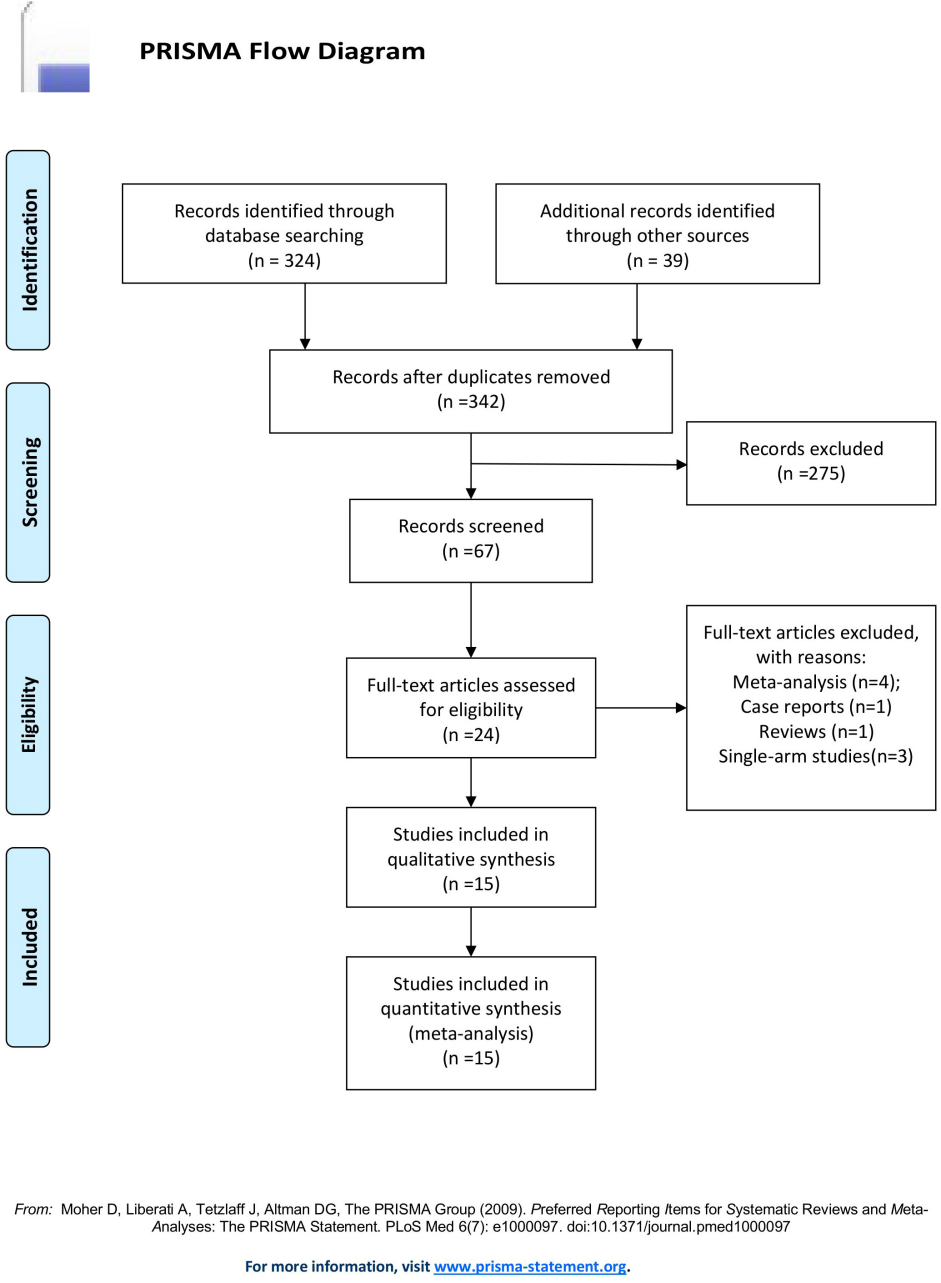
PRISMA flow diagram.

A total of 363 articles were collected. The duplicates were excluded; then 342 articles remained. Then, reviews, case reports, and other types of articles were excluded. Finally, 15 articles remained (21–35). The detailed steps of our literature search are shown in **Figure 1**. Fifteen studies with a total of 1365 patients were included in the final analysis. A total of 688 patients (50.40%) received TACE+L+I, and 677 (49.60%) patients received TACE+L. The characteristics of these studies are listed in **Table 1**.

**Table 1:** Basic characteristics and quality assessment of included studies.

Study	Country	Study period	Study type	Case	Gender(F/M)		Age		Quality*
				TACE+L+I vs. TACE+L	TACE+L+I	TACE+L	TACE+L+I	TACE+L	
Cai M, et al., 2022 (21)	China	2019-2020	R	41 vs. 40	4/37	7/33	51.9 ± 10.3	54.6 ± 11.0	7
Chen S, et al., 2024 (22)	China	2016-2020	R	70 vs. 72	37/33	38/34	NA	NA	7
Ding ZR, et al., 2023 (23)	China	2019-2022	R	19 vs. 16	1/18	3/13	57.0 [47.0, 64.5]	61.5 [53.0, 65.0]	7
Guo P, et al., 2022 (24)	China	2018-2022	R	48 vs. 48	1/47	2/46	NA	NA	6
Jiang J, et al., 2023 (25)	China	2018-2022	R	42 vs. 45	9/33	4/41	61.71 ± 9.48	61.24 ± 12.10	7
Qu WF, et al., 2022 (26)	China	2018-2021	R	30 vs. 21	4/26	1/20	55.5 (47.8, 64.3)	50.0 (45.0, 61.0)	6
Sun B, et al., 2022 (27)	China	2018-2021	R	31 vs. 52	6/25	6/46	54.84 ± 9.249	51.77 ± 9.791	7
Wang WJ, et al., 2023 (28)	China	2019-2020	R	51 vs. 45	5/49	2/43	57.0 ± 9.9	60.8 ± 9.4	7
Wang YY, et al., 2023 (29)	China	2017-2022	R	45 vs. 20	3/42	5/15	54 (18-79)	62 (26-75)	7
Wu XH, et al., 2024 (30)	China	2019-2020	R	18 vs. 23	3/15	5/18	56.9 ± 8.1	58.1 ± 9.4	7
Xiang Z, et al., 2023 (31)	China	2018-2021	R	33 vs. 49	5/28	4/45	51.0 ± 12.2	51.7 ± 11.2	7
Yang H, et al.2023 (32)	China	2019-2022	R	64 vs. 58	13/64	11/58	61.4 ± 9.3	63.2 ± 8.5	7
Zhao S, et al., 2022 (33)	China	2018-2020	R	23 vs. 32	0/23	1/31	52.83 ± 7.14	57.38 ± 9.44	7
Zhao YS, et al., 2024 (34)	China	2021-2023	R	103 vs. 66	24/78	7/59	NA	NA	7
Zou X, et al., 2023 (35)	China	2018-2022	R	70 vs. 90	11/59	13/77	53.6 ± 15.1	52.3 ± 14.8	7

R, Retrospective cohort studies; M, Male; F, Female; TACE, Transarterial chemoembolization; L, lenvatinib; I, immune checkpoint inhibitors; TACE+L, transarterial chemoembolization combined with lenvatinib; TACE+L+P, transarterial chemoembolization combined with lenvatinib plus programmed death 1 and programmed death ligand 1 inhibitors; HBV, Hepatitis B virus; ECOG, Eastern Cooperative Oncology Group; AFP, Alpha fetoprotein; NA, not available; *: Quality assessment of included studies was based on the Newcastle-Ottawa Scale, and it is shown in **Supplementary File S2**.

### 3.2 Meta-analysis results

The treatment effects of TACE+L+I and TACE+L were compared by assessing tumor response, long-term survival outcome, and adverse events. The results are listed in **Tables 2**, **3**.

**Table 2:** Tumor response rate and long-term outcome.

Measured Outcomes	No. Studies	Heterogeneity Test		Model	RR/HR	95%CI	*P*
		*I^2^* (%)	*P*				
Complete response	15	0	0.94	Random	2.34	1.53,3.59	**<0.0001**
Partial response	15	0	0.73	Random	1.45	1.28,1.64	**<0.0001**
Stable disease	15	30	0.13	Random	0.85	0.69,1.03	0.10
Progressive disease	15	8	0.36	Random	0.39	0.30,0.51	**<0.00001**
Objective response rate	15	0	0.51	Random	1.55	1.39,1.73	**<0.00001**
Disease control rate	15	83	<0.00001	Random	1.22	1.10,1.36	**0.0003**
Overall survival	10	5.4	0.392	Random	2.32	1.95, 2.75	**<0.05**
Progression free survival	13	67.8	0	Random	2.30	1.80, 2.93	**<0.05**

RR/HR, relative risk/hazard ratio; CI, confidence interval; CR, complete response; PR, partial response; SD, stable disease; PD, progressive disease; ORR (CR+PR), objective response rate; DCR (CR+PR+SD), disease control rate; P-Values less than 0.05 are shown in bold.

**Table 3:** Adverse events.

Adverse events	Grade	No. Studies	Heterogeneity Test		Model	RR	95%CI	*P*
			*I^2^* (%)	*P*				
Hypertension	Any Grade	15	0	0.97	Random	1.19	1.00,1.41	0.05
Diarrhea	Any Grade	12	0	1	Random	1.18	0.95,1.46	0.14
Hand-foot syndrome	Any Grade	13	0	0.97	Random	1.04	0.87,1.24	0.7
Fatigue	Any Grade	12	0	0.97	Random	1.1	0.93,1.30	0.28
Elevated AST	Any Grade	6	0	0.55	Random	1.02	0.92,1.13	0.72
Elevated ALT	Any Grade	7	0	0.87	Random	1.08	0.93,1.26	0.31
Decreased appetite	Any Grade	9	0	0.98	Random	0.98	0.71,1.37	0.92
Hypothyroidism	Any Grade	11	0	0.84	Random	1.81	1.20,2.71	**0.004**
Abdominal pain	Any Grade	12	9	0.36	Random	1.04	0.91,1.18	0.55
Thrombocytopenia	Any Grade	6	0	0.98	Random	1.16	0.81,1.66	0.43
Rash	Any Grade	9	0	0.85	Random	1.13	0.92,1.39	0.24
Nausea	Any Grade	13	0	0.90	Random	1.00	0.85,1.17	1

RR, relative risk; CI, confidence interval; No.: number of; P-Values less than 0.05 are shown in bold.

#### 3.2.1 Tumor response

Fifteen studies (21–35) reported complete response, partial response, stable disease, progression of disease, objective response rate, and disease control rate. The meta-analysis showed that the TACE+L+I group was significantly better than the TACE+L group in complete response (RR = 2.34, 95%CI:1.53, 3.59, *p* < 0.0001), partial response (RR = 1.45, 95%CI:1.28, 1.64, *p* < 0.0001), progression of disease (RR = 0.39, 95%CI:0.30, 0.51, *p* < 0.00001), objective response rate (RR = 1.55, 95%CI:1.39, 1.73, *p* < 0.00001), disease control rate (RR = 1.22, 95%CI:1.10, 1.36, *p* = 0.0003), whereas the TACE+L+I group was not significantly different from the TACE+L group in stable disease (RR = 0.85, 95%CI:0.69, 1.03, *p* = 0.10). As shown in **Table 2**.

#### 3.2.2 Long-term survival outcome

**Figure 2:**
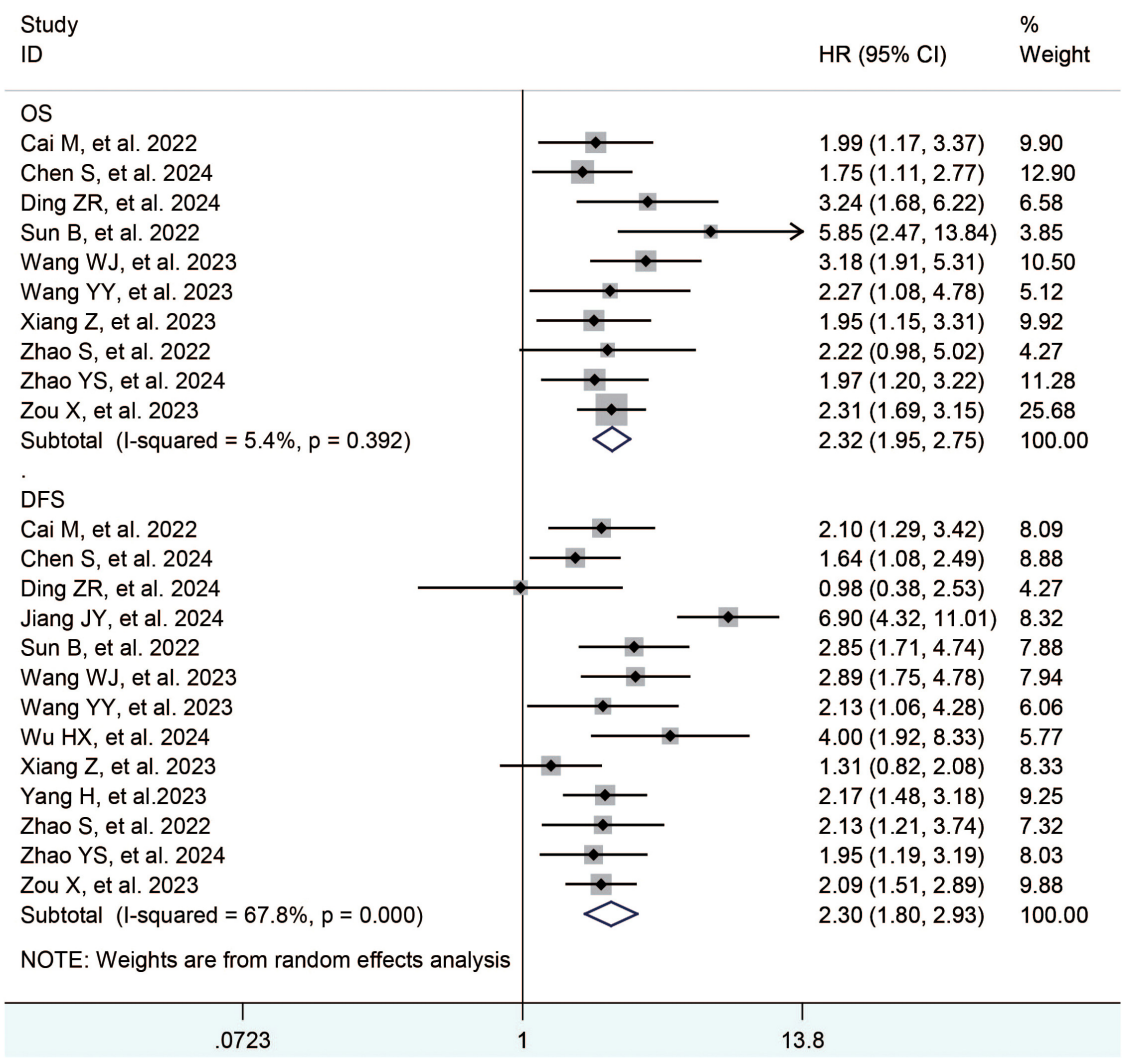
Forest plots survival (OS) and disease-free survival.

Thirteen studies (23, 25, 27–35) reported the overall survival and progression free survival. The meta-analysis showed that the TACE+L+I group was significantly better than the TACE+L group in terms of overall survival (HR = 2.32, 95%CI:1.95, 3.15, *p* < 0.05) and progression free survival (HR = 2.30, 95%CI:1.80, 2.93, *p* < 0.05), as shown in **Table 2** and **Figure 2**.

##### 3.2.2.1 Adverse events

**Figure 3:**
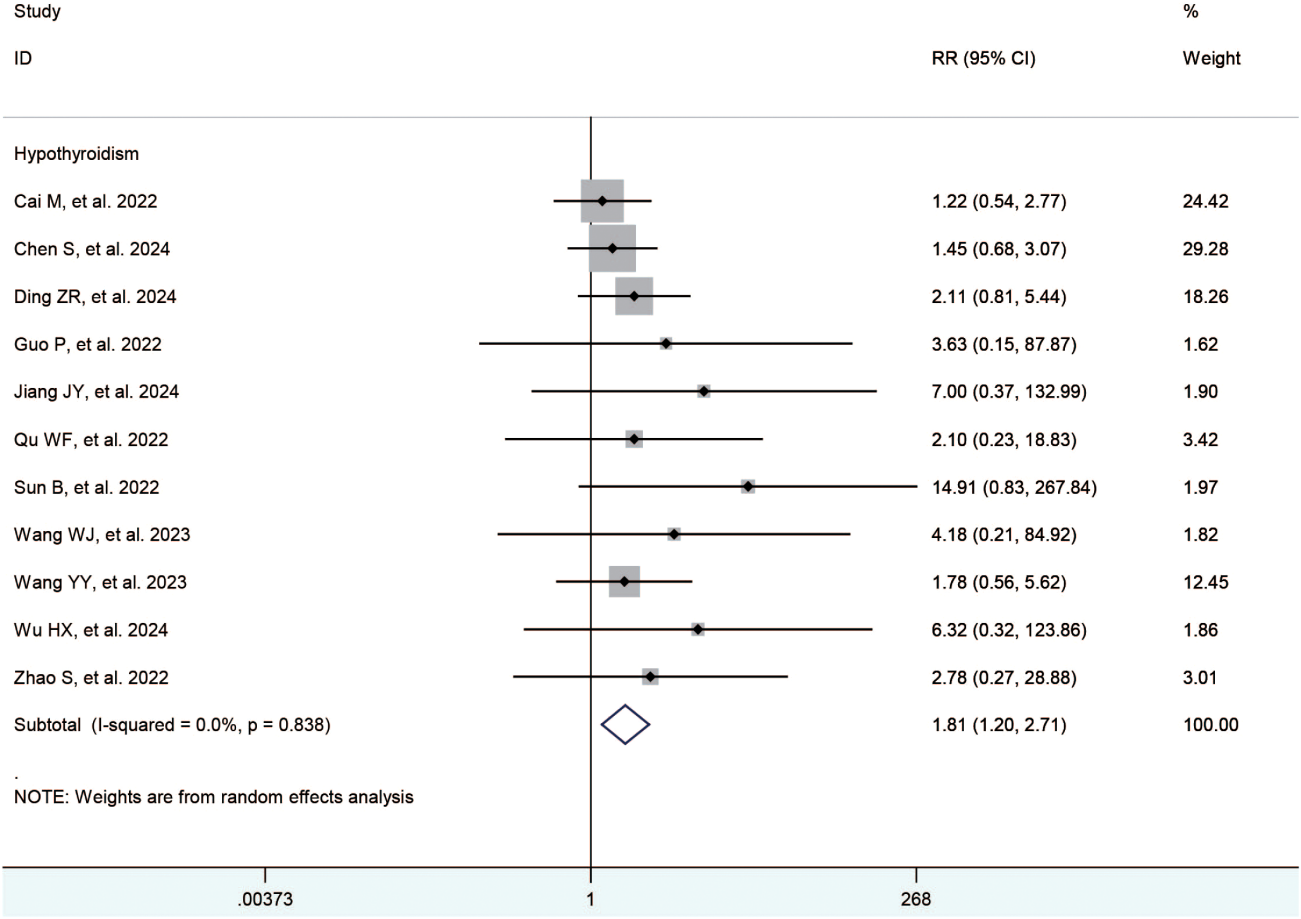
Forest plots of the incidence of hypothyroidism.

The included studies (21–35) reported the hypertension, diarrhea, hand-foot syndrome, fatigue, elevated ast, elevated alt, decreased appetite, hypothyroidism, abdominal pain, thrombocytopenia, rash, and nausea. The meta-analysis showed no significant difference between the TACE+L+I and TACE+L group regarding hypertension (RR = 1.19, 95%CI:1.00, 1.41, *P* = 0.05), diarrhea (RR = 1.18, 95%CI:0.95,1.46, *P* = 0.14), hand-foot syndrome (RR = 1.04, 95%CI:0.87,1.24, *P* = 0.70), fatigue (RR = 1.10, 95%CI:0.93, 1.30, *P* = 0.28), elevated AST (RR = 1.02, 95%CI:0.92,1.13, *P* = 0.72), elevated ALT (RR = 1.08, 95%CI:0.93,1.26, *P* = 0.31), decreased appetite (RR = 0.98, 95%CI:0.71,1.37, *P* = 0.92), abdominal pain (RR = 1.04, 95%CI:0.91, 1.18, *P* = 0.55), thrombocytopenia (RR = 1.16, 95% CI:0.81,1.66, *P* = 0.43), rash (RR = 1.13, 95%CI. 0.92, 1.39, *P* = 0.24), and nausea (RR = 1.00, 95%CI:0.85,1.17, *P* = 1.00). However, hypothyroidism was significantly higher in the TACE+L+I group (RR = 1.81, 95%CI: 1.20, 2.71, *P* = 0.004) than in the TACE+L group, as shown in **Table 3** and **Figure 3**.

### 3.3 Sensitivity analysis and publication bias

The results of each meta-analysis were stable when the sensitivity analysis was performed. Begg’s funnel plot with pseudo 95% confidence limits is shown in **Supplementary File S3**. Publication bias was not found using Begg’s test and Egger’s test, as shown in **Supplementary File S4**.

## 4 Discussion

HCC is the fifth most common cancer worldwide and the second most common cause of cancer-related deaths (1). First-line treatment options for HCC include surgical resection, ablation, liver transplantation, TACE, and drugs, but despite that, the prognosis of uHCC remains poor (36). The TACE is the standard treatment recommended by the European Association for the Study of the Liver guidelines and the Barcelona Clinic Liver Cancer (BCLC) treatment strategy for intermediate and advanced HCC (37, 38), and it may result in tumor response in up to 50% of HCC, resulting in a survival benefit. However, TACE alone has limited therapeutic efficacy as a local-regional therapy, which may promote anti-tumor immunity by releasing tumor antigens and inducing damage-associated molecules that induce “immunogenic cell death” (39, 40). Furthermore, the hypoxic microenvironment after TACE may result in the expression of VEGF and PD-L1 (39–42). Antiangiogenic drugs combined with ICIs represent a promising addition to TACE (43–48). So far, the conclusions of various studies regarding the survival benefit from TACE+L+I of patients with uHCC are inconsistent.

The results of our meta-analysis found that complete response, partial response, objective response rate, and disease control rate were significantly higher in the TACE+L+I group, while progression of disease was significantly lower in the TACE+L+I group than in the TACE+L group, suggesting that the addition of ICIs has a significantly enhanced the tumor response rate. Different therapeutic approaches at different clinical stages are needed to combat HCC, and combination therapy may be used (49, 50). Although tumor response rates suggest that the TACE+L+I is superior to the TACE+L, the effectiveness of antitumor treatments should be based on more direct evidence of clinical benefit, such as prolonged survival, improved quality of life, or reduction of associated symptoms. These clinical benefits are sometimes not predicted by tumor response rates. Therefore, the survival data were analyzed, revealing that the overall survival and progression-free survival were longer in the TACE+L+I group than in the TACE+L group.

Currently, the combination of TACE and lenvatinib is considered clinically safe. Our meta-analysis found that the TACE+L+I group had a significantly higher incidence of hypothyroidism compared to the TACE+L group, while the TACE+L+I group was not significantly different from the TACE+L group in other adverse events, such as hypertension, diarrhea, hand-foot syndrome, fatigue, elevated ast, elevated alt, decreased appetite, hypothyroidism, abdominal pain, thrombocytopenia, and rash, nausea. Common adverse events with lenvatinib included hand and foot skin reactions, diarrhea, and hypertension, while common adverse events with TACE included pain and transient transaminase elevations. Fortunately, treatment-related adverse events were predominantly grade 1 or 2 and were resolved or eliminated after appropriate and prompt management. Therefore, the adverse events associated with TACE+L+I were acceptable.

Several meta-analyses are available on similar topics, but all of them have limitations. Gao Y, et al. (51) performed a meta-analysis and concluded that TACE or hepatic arterial infusion chemotherapy (HAIC) combined with lenvatinib plus PD-1 inhibitor could effectively delays the progression of HCC, prolong the survival, and improve the quality of life of HCC patients with portal vein thrombosis. The intervention in that study included HAIC in addition to TACE. In another meta-analysis by Liu J, et al. (52), the authors compared the efficacy of TACE plus tyrosine kinase inhibitors and ICIs (T+T+I) with that of TACE plus tyrosine kinase inhibitors (T+T) for the treatment of uHCC, concluding that T+T+I for advanced HCC had better objective response rate, as well as longer progression-free survival and overall survival than TACE+T, with no significant increase in adverse events. They did not distinguish between lenvatinib and other tyrosine kinase inhibitors in the study, making the conclusions too broadly applicable. The first systematic review on T+L+I for uHCC was conducted by Sun L, et al. (53), but no further meta-analysis was performed. The first meta-analysis on the same topic was conducted by Liu J, et al. (54) in 2023. Eight cohort studies on TACE plus lenvatinib with or without ICIs for uHCC were included. The T+L+I group had significantly longer overall and progression-free survival in that meta-analysis, as well as higher objective response and disease control rates, which was consistent with our findings; however, there was a higher incidence of hypertension, vomiting or nausea, and hypothyroidism in the T+L+I group, which was inconsistent with our study. Our meta-analysis only found that the T+L+I group was significantly higher than the T+L group in hypothyroidism. The conclusions of our meta-analysis may be more in line with the first-line clinic, as the latest studies we included allowed for a larger number of patients to reduce the potential bias in previous meta-analyses.

This is the most comprehensive meta-analysis available assessing TACE+L+I for uHCC, and although most of the included studies were retrospective, which would be inherently subjected to selection and publication bias, retrospective studies can be reflective of the real world. Our findings assessed the real-world clinical efficacy of TACE+L+I for the treatment of uHCC, and provided a guidance for subsequent clinical studies, although this combination therapy needs further exploration in future randomized controlled trials.

## 5 Conclusions

TACE+L+I for advanced HCC resulted in significantly better tumor response rates, overall survival, and disease-free survival than TACE+L, while the incidence of hypothyroidism was higher in the TACE+L+I group than in the TACE+L group. The adverse events of ICIs were acceptable compared to the survival benefit of ICIs. These conclusions still need to be further confirmed in the future with high-quality randomized controlled trials.

## 6 Limitations

Our study has several limitations. First, this was a retrospective study, which may lead to selection bias. Second, there was significant heterogeneity across the included studies in terms of disease control rate. By exploring the source of the heterogeneity, we found that it was not originated from a particular study or studies, which may be related to the data distribution characteristics, because disease control rate corresponds to progressive disease, and inter-study heterogeneity is low in progressive disease. Therefore, subsequent scholars should be cautious in applying the outcome. Finally, most of the included studies were from a single institution with a limited number of cases and most came from China, leading to conclusions with little explanatory power.

## Data Availability

The original contributions presented in the study are included in the article/**Supplementary Material**. Further inquiries can be directed to the corresponding authors.

## References

[1] Sung H Ferlay J Siegel RL Laversanne M Soerjomataram I Jemal A . Global cancer statistics 2020: GLOBOCAN estimates of incidence and mortality worldwide for 36 cancers in 185 countries. CA Cancer J Clin. (2021) 71:209–49. doi: 10.3322/caac.21660 33538338

[2] Zhou J Sun H Wang Z Cong W Wang J Zeng M . Guidelines for the diagnosis and treatment of hepatocellular carcinoma (2019 edition). Liver Cancer. (2020) 9:682–720. doi: 10.1159/000509424 33442540 PMC7768108

[3] Benson AB D’Angelica MI Abbott DE Anaya DA Anders R Are C . Hepatobiliary cancers, version 2.2021, NCCN clinical practice guidelines in oncology. J Natl Compr Canc Netw. (2021) 19:541–65. doi: 10.6004/jnccn.2021.0022 34030131

[4] European Association For The Study Of The Liver . Corrigendum to ‘EASL recommendations on treatment of hepatitis C: Final update of the series^’^ [J Hepatol 73 (2020) 1170-1218. J Hepatol. (2023) 78:452. doi: 10.1016/j.jhep.2022.10.006 36464532

[5] Fu Z Li X Zhong J Chen X Cao K Ding N . Lenvatinib in combination with transarterial chemoembolization for treatment of unresectable hepatocellular carcinoma (uHCC): a retrospective controlled study. Hepatol Int. (2021) 15:663–75. doi: 10.1007/s12072-021-10184-9 PMC8286947 33877527

[6] Chang Y Jeong SW Young Jang J Jae Kim Y . Recent updates of transarterial chemoembolilzation in hepatocellular carcinoma. Int J Mol Sci. (2020) 21:8165. doi: 10.3390/ijms21218165 33142892 PMC7662786

[7] Kishore SA Bajwa R Madoff DC . Embolotherapeutic strategies for hepatocellular carcinoma: 2020 update. Cancers (Basel). (2020) 12(4):791. doi: 10.3390/cancers12040791 32224882 PMC7226474

[8] Kudo M Finn RS Qin S Han KH Ikeda K Piscaglia F . Lenvatinib versus sorafenib in first-line treatment of patients with unresectable hepatocellular carcinoma: a randomised phase 3 non-inferiority trial. Lancet. (2018) 391:1163–73. doi: 10.1016/S0140-6736(18)30207-1 29433850

[9] Chen S Wu Z Shi F Mai Q Wang L Wang F . Lenvatinib plus TACE with or without pembrolizumab for the treatment of initially unresectable hepatocellular carcinoma harbouring PD-L1 expression: a retrospective study. J Cancer Res Clin Oncol. (2022) 148:2115–25. doi: 10.1007/s00432-021-03767-4 PMC9293824 34453221

[10] Kudo M . A new treatment option for intermediate-stage hepatocellular carcinoma with high tumor burden: initial lenvatinib therapy with subsequent selective TACE. Liver Cancer. (2019) 8:299–311. doi: 10.1159/000502905 31768341 PMC6872999

[11] Yuan P Song J Wang F Zhu G Chen B . Combination of TACE and Lenvatinib as a promising option for downstaging to surgery of initially unresectable intrahepatic cholangiocarcinoma. Invest New Drugs. (2022) 40:1125–32. doi: 10.1007/s10637-022-01257-z 35793038

[12] Cao F Yang Y Si T Luo J Zeng H Zhang Z . The efficacy of TACE combined with lenvatinib plus sintilimab in unresectable hepatocellular carcinoma: A multicenter retrospective study. Front Oncol. (2021) 11:783480. doi: 10.3389/fonc.2021.783480 34988019 PMC8721033

[13] Peng Z Fan W Zhu B Wang G Sun J Xiao C . Lenvatinib combined with transarterial chemoembolization as first-line treatment for advanced hepatocellular carcinoma: A phase III, randomized clinical trial (LAUNCH). J Clin Oncol. (2023) 41:117–27. doi: 10.1200/JCO.22.00392 35921605

[14] Cheng AL Hsu C Chan SL Choo SP Kudo M . Challenges of combination therapy with immune checkpoint inhibitors for hepatocellular carcinoma. J Hepatol. (2020) 72:307–19. doi: 10.1016/j.jhep.2019.09.025 31954494

[15] Finn RS Ikeda M Zhu AX Sung MW Baron AD Kudo M . Phase ib study of lenvatinib plus pembrolizumab in patients with unresectable hepatocellular carcinoma. J Clin Oncol. (2020) 38:2960–70. doi: 10.1200/JCO.20.00808 PMC7479760 32716739

[16] Qu S Zhang X Wu Y Meng Y Pan H Fang Q . Efficacy and safety of TACE combined with lenvatinib plus PD-1 inhibitors compared with TACE alone for unresectable hepatocellular carcinoma patients: A prospective cohort study. Front Oncol. (2022) 12:874473. doi: 10.3389/fonc.2022.874473 35530353 PMC9068979

[17] Guo X Nie H Zhang W Li J Ge J Xie B . Contrasting cytotoxic and regulatory T cell responses underlying distinct clinical outcomes to anti-PD-1 plus lenvatinib therapy in cancer. Cancer Cell. (2025) 43:248–68. doi: 10.1016/j.ccell.2025.01.001 39889705

[18] Kimura T Kato Y Ozawa Y Kodama K Ito J Ichikawa K . Immunomodulatory activity of lenvatinib contributes to antitumor activity in the Hepa1–6 hepatocellular carcinoma model. Cancer Sci. (2018) 109:3993–4002. doi: 10.1111/cas.2018.109.issue-12 30447042 PMC6272102

[19] Yi C Chen L Lin Z Liu L Shao W Zhang R . Lenvatinib targets FGF receptor 4 to enhance antitumor immune response of anti-programmed cell death-1 in HCC. Hepatology. (2021) 74:2544–60. doi: 10.1002/hep.31921 34036623

[20] Liberati A Altman DG Tetzlaff J Mulrow C Gøtzsche PC Ioannidis JP . The PRISMA statement for reporting systematic reviews and meta-analyses of studies that evaluate health care interventions: explanation and elaboration. J Clin Epidemiol. (2009) 62:e1–34. doi: 10.1016/j.jclinepi.2009.06.006 19631507

[21] Cai M Huang W Huang J Shi W Guo Y Liang L . Transarterial chemoembolization combined with lenvatinib plus PD-1 inhibitor for advanced hepatocellular carcinoma: A retrospective cohort study. Front Immunol. (2022) 13:848387. doi: 10.3389/fimmu.2022.848387 35300325 PMC8921060

[22] Chen S Shuangyan T Shi F Cai H Wu Z Wang L . TACE plus lenvatinib and tislelizumab for intermediate-stage hepatocellular carcinoma beyond up-to-11 criteria: a multicenter cohort study. Front Immunol. (2024) 15:1430571. doi: 10.3389/fimmu.2024.1430571 39131156 PMC11310062

[23] Ding Z Fang G Tang Y Zeng Y . The impact of PD-1 inhibitors on prognosis in unresectable hepatocellular carcinoma treated with TACE and lenvatinib: a retrospective study. Sci Rep. (2024) 14:14334. doi: 10.1038/s41598-024-63571-1 38906915 PMC11192886

[24] Guo P Pi X Gao F Li Q Li D Feng W . Transarterial chemoembolization plus lenvatinib with or without programmed death-1 inhibitors for patients with unresectable hepatocellular carcinoma: A propensity score matching study. Front Oncol. (2022) 12:945915. doi: 10.3389/fonc.2022.945915 36338683 PMC9630329

[25] Jiang J Zhang H Lai J Zhang S Ou Y Fu YN Efficacy and safety of transarterial chemoembolization plus lenvatinib with or without tislelizumab as the first-Line treatment for unresectable hepatocellular carcinoma: a propensity score matching analysis. J Hepatocell Carcinoma. (2024) 1:1607–22. doi: 10.2147/JHC.S472286 PMC11352531 39206422

[26] Qu WF Ding ZB Qu XD Tang Z Zhu GQ Fu XT . Conversion therapy for initially unresectable hepatocellular carcinoma using a combination of toripalimab, lenvatinib plus TACE: real-world study. BJS Open. (2022) 6:zrac114. doi: 10.1093/bjsopen/zrac114 36125345 PMC9499852

[27] Sun B Zhang L Sun T Ren Y Cao Y Zhang W . Safety and efficacy of lenvatinib combined with camrelizumab plus transcatheter arterial chemoembolization for unresectable hepatocellular carcinoma: A two-center retrospective study. Front Oncol. (2022) 12:982948. doi: 10.3389/fonc.2022.982948 36172158 PMC9511022

[28] Wang WJ Liu ZH Wang K Yu HM Cheng YQ Xiang YJ . Efficacy and safety of TACE combined with lenvatinib and PD-1 inhibitors for unresectable recurrent HCC: A multicenter, retrospective study. Cancer Med. (2023) 12:11513–24. doi: 10.1002/cam4.v12.10 PMC10242311 36999793

[29] Wang YY Yang X Wang YC Long JY Sun HS Li YR . Clinical outcomes of lenvatinib plus transarterial chemoembolization with or without programmed death receptor-1 inhibitors in unresectable hepatocellular carcinoma. World J Gastroenterol. (2023) 29:1614–26. doi: 10.3748/wjg.v29.i10.1614 PMC10037246 36970591

[30] Wu HX Ding XY Xu YW Yu MH Li XM Deng N . Transcatheter arterial chemoembolization combined with PD-1 inhibitors and Lenvatinib for hepatocellular carcinoma with portal vein tumor thrombus. World J Gastroenterol. (2024) 30:843–54. doi: 10.3748/wjg.v30.i8.843 PMC10950640 38516240

[31] Xiang Z Li G Mu L Wang H Zhou C Yan H . and camrelizumab for unresectable multiple nodular and large hepatocellular carcinoma (>5 cm). Technol Cancer Res Treat. (2023) 22:15330338231200320. doi: 10.1177/15330338231200320 37723998 PMC10510362

[32] Yang H Yang T Qiu G Liu J . Efficacy and safety of TACE combined with lenvatinib and PD-(L)1 inhibitor in the treatment of unresectable hepatocellular carcinoma: A retrospective study. J Hepatocell Carcinoma. (2023) :10:1435–1443. doi: 10.2147/JHC.S423684 PMC10492540 37691972

[33] Zhao S Zhou M Wang P Yang J Zhang D Yin F . Sorafenib, lenvatinib, or lenvatinib combining PD-1 inhibitors plus TACE in unresectable hepatocellular carcinoma: A retrospective analysis. Technol Cancer Res Treat. (2022) 21:15330338221133640. doi: 10.1177/15330338221133640 36259214 PMC9583225

[34] Zhao Y Wen S Xue Y Dang Z Nan Z Wang D . Transarterial chemoembolization combined with lenvatinib plus tislelizumab for unresectable hepatocellular carcinoma: a multicenter cohort study. Front Immunol. (2024) 15:1449663. doi: 10.3389/fimmu.2024.1449663 39411718 PMC11473327

[35] Zou X Xu Q You R Yin G . Correlation and efficacy of TACE combined with lenvatinib plus PD-1 inhibitor in the treatment of hepatocellular carcinoma with portal vein tumor thrombus based on immunological features. Cancer Med. (2023) 12:11315–33. doi: 10.1002/cam4.v12.10 PMC10242346 36951443

[36] Lau WY Leung TW Lai BS Liew CT Ho SK Yu SC . Preoperative systemic chemoimmunotherapy and sequential resection for unresectable hepatocellular carcinoma. Ann Surg. (2001) 233:236–41. doi: 10.1097/00000658-200102000-00013 PMC1421206 11176130

[37] Bruix J Sherman M American Association for the Study of Liver Diseases . Management of hepatocellular carcinoma: an update. Hepatology. (2011) 53:1020–2. doi: 10.1002/hep.24199 PMC3084991 21374666

[38] Forner A Reig M Bruix J . Hepatocellular carcinoma. Lancet. (2018) 391:1301–14. doi: 10.1016/S0140-6736(18)30010-2 29307467

[39] Galluzzi L Buqué A Kepp O Zitvogel L Kroemer G . Immunogenic cell death in cancer and infectious disease. Nat Rev Immunol. (2017) 17:97–111. doi: 10.1038/nri.2016.107 27748397

[40] Zhou J Sun HC Wang Z Cong WM Wang JH Zeng MS . Guidelines for diagnosis and treatment of primary liver cancer in China (2017 edition). Liver Cancer. (2018) 7:235–60. doi: 10.1159/000488035 PMC6167671 30319983

[41] Sergio A Cristofori C Cardin R Pivetta G Ragazzi R Baldan A . Transcatheter arterial chemoembolization (TACE) in hepatocellular carcinoma (HCC): the role of angiogenesis and invasiveness. Am J Gastroenterol. (2008) 103:914–21. doi: 10.1111/j.1572-0241.2007.01712.x 18177453

[42] Pinato DJ Murray SM Forner A Kaneko T Fessas P Toniutto P . Trans-arterial chemoembolization as a loco-regional inducer of immunogenic cell death in hepatocellular carcinoma: implications for immunotherapy. J Immunother Cancer. (2021) 9:e003311. doi: 10.1136/jitc-2021-003311 34593621 PMC8487214

[43] Pinato DJ Howell J Ramaswami R Sharma R . Review article: delivering precision oncology in intermediate-stage liver cancer. Aliment Pharmacol Ther. (2017) 45:1514–23. doi: 10.1111/apt.2017.45.issue-12 28440552

[44] Kudo M Imanaka K Chida N Nakachi K Tak WY Takayama T . Phase III study of sorafenib after transarterial chemoembolisation in Japanese and Korean patients with unresectable hepatocellular carcinoma. Eur J Cancer. (2011) 47:2117–27. doi: 10.1016/j.ejca.2011.05.007 21664811

[45] Kudo M Han G Finn RS Poon RT Blanc JF Yan L . Brivanib as adjuvant therapy to transarterial chemoembolization in patients with hepatocellular carcinoma: A randomized phase III trial. Hepatology. (2014) 60:1697–707. doi: 10.1002/hep.27290 24996197

[46] Meyer T Fox R Ma YT Ross PJ James MW Sturgess R . Sorafenib in combination with transarterial chemoembolisation in patients with unresectable hepatocellular carcinoma (TACE 2): a randomised placebo-controlled, double-blind, phase 3 trial. Lancet Gastroenterol Hepatol. (2017) 2:565–75. doi: 10.1016/S2468-1253(17)30156-5 28648803

[47] Kudo M Cheng AL Park JW Park JH Liang PC Hidaka H . Orantinib versus placebo combined with transcatheter arterial chemoembolisation in patients with unresectable hepatocellular carcinoma (ORIENTAL): a randomised, double-blind, placebo-controlled, multicentre, phase 3 study. Lancet Gastroenterol Hepatol. (2018) 3:37–46. doi: 10.1016/S2468-1253(17)30290-X 28988687

[48] Park JW Kim YJ Kim DY Bae SH Paik SW Lee YJ . Sorafenib with or without concurrent transarterial chemoembolization in patients with advanced hepatocellular carcinoma: The phase III STAH trial. J Hepatol. (2019) 70:684–91. doi: 10.1016/j.jhep.2018.11.029 30529387

[49] Xiang YJ Wang K Yu HM Li XW Cheng YQ Wang WJ . Transarterial chemoembolization plus a PD-1 inhibitor with or without lenvatinib for intermediate-stage hepatocellular carcinoma. Hepatol Res. (2022) 52:721–9. doi: 10.1111/hepr.13773 35536197

[50] Bruix J Gores GJ Mazzaferro V . Hepatocellular carcinoma: clinical frontiers and perspectives. Gut. (2014) 63:844–55. doi: 10.1136/gutjnl-2013-306627 PMC4337888 24531850

[51] Yuanren G Yin L Liu R Cui Y . The treatment of transarterial chemoembolization/hepatic arterial infusion chemotherapy combined with lenvatinib and PD-1 inhibitor is effective against hepatocellular carcinoma with portal vein tumor thrombus: A systematic review. Front Oncol. (2023) 13:1054072. doi: 10.3389/fonc.2023.1054072 36969065 PMC10034403

[52] Liu J Wang P Shang L Zhang Z Tian Y Chen X . TACE plus tyrosine kinase inhibitors and immune checkpoint inhibitors versus TACE plus tyrosine kinase inhibitors for the treatment of patients with hepatocellular carcinoma: a meta-analysis and trial sequential analysis. Hepatol Int. (2024) 18:595–609. doi: 10.1007/s12072-023-10591-0 37843788

[53] Sun L Xu X Meng F Liu Q Wang H Li X . Lenvatinib plus transarterial chemoembolization with or without immune checkpoint inhibitors for unresectable hepatocellular carcinoma: A review. Front Oncol. (2022) 12:980214. doi: 10.3389/fonc.2022.980214 36249023 PMC9555078

[54] Liu J Wei S Yang L Yu J Yan D Yi P . Efficacy and safety of transarterial chemoembolization plus lenvatinib with or without programmed death-1 inhibitors in the treatment of unresectable hepatocellular carcinoma: a systematic review and meta-analysis. J Cancer Res Clin Oncol. (2023) 149:14451–61. doi: 10.1007/s00432-023-05231-x PMC11797731 37563417

