# Supplementary material for: The impact of immune checkpoint inhibitors on prognosis in unresectable hepatocellular carcinoma treated with TACE and lenvatinib: a meta-analysis
Source: Front Immunol. 2025 May 21;16:1573505. doi: 10.3389/fimmu.2025.1573505 (PMC12133757; doi:10.3389/fimmu.2025.1573505)
Supplement: Supplementary file 1 [file DataSheet1.zip › Supplementary files/Supplementary file 1. Search strategy.docx]

**Supplementary file 1. Search strategy**

**Pubmed：**

**(("Immune Checkpoint Inhibitors"[Mesh] OR "Immune Checkpoint Inhibitors" [Pharmacological Action] OR "immune checkpoint inhibitor BMS-1" [Supplementary Concept]) OR (immune checkpoint inhibitor)) AND ((("chemoembolization, therapeutic"[MeSH Terms] AND (("transarterial"[All Fields] OR "transarterially"[All Fields]) AND ("chemoembolic"[All Fields] OR "chemoembolisation"[All Fields] OR "chemoembolisations"[All Fields] OR "chemoembolism"[All Fields] OR "chemoembolization"[All Fields] OR "chemoembolizations"[All Fields] OR "chemoembolized"[All Fields]))) OR "TACE"[All Fields]) AND ("lenvatinib"[Supplementary Concept] OR ("lenvatinib"[Supplementary Concept] OR "lenvatinib"[All Fields])) AND ("carcinoma, hepatocellular"[MeSH Terms] OR ("carcinoma, hepatocellular"[MeSH Terms] OR ("carcinoma"[All Fields] AND "hepatocellular"[All Fields]) OR "hepatocellular carcinoma"[All Fields] OR ("hepatocellular"[All Fields] AND "carcinoma"[All Fields]))))**

("Immune Checkpoint Inhibitors"[MeSH Terms] OR "Immune Checkpoint Inhibitors"[Pharmacological Action] OR "immune checkpoint inhibitor BMS-1"[Supplementary Concept] OR ("Immune Checkpoint Inhibitors"[Pharmacological Action] OR "Immune Checkpoint Inhibitors"[MeSH Terms] OR ("immune"[All Fields] AND "checkpoint"[All Fields] AND "inhibitors"[All Fields]) OR "Immune Checkpoint Inhibitors"[All Fields] OR ("immune"[All Fields] AND "checkpoint"[All Fields] AND "inhibitor"[All Fields]) OR "immune checkpoint inhibitor"[All Fields])) AND ((("chemoembolization, therapeutic"[MeSH Terms] AND (("transarterial"[All Fields] OR "transarterially"[All Fields]) AND ("chemoembolic"[All Fields] OR "chemoembolisation"[All Fields] OR "chemoembolisations"[All Fields] OR "chemoembolism"[All Fields] OR "chemoembolization"[All Fields] OR "chemoembolizations"[All Fields] OR "chemoembolized"[All Fields]))) OR "TACE"[All Fields]) AND ("lenvatinib"[Supplementary Concept] OR ("lenvatinib"[Supplementary Concept] OR "lenvatinib"[All Fields])) AND ("carcinoma, hepatocellular"[MeSH Terms] OR ("carcinoma, hepatocellular"[MeSH Terms] OR ("carcinoma"[All Fields] AND "hepatocellular"[All Fields]) OR "hepatocellular carcinoma"[All Fields] OR ("hepatocellular"[All Fields] AND "carcinoma"[All Fields]))))

**Embase:**

Session Results

.......................................................

No. Query Results Results

#9. #3 AND #4 AND #7 AND #8 250

#8. 'immune checkpoint inhibitor'/exp OR 'immune 47,325

checkpoint inhibitor'

#7. #5 OR #6 31,851

#6. 'tace'/exp OR tace 15,809

#5. 'chemoembolization'/exp OR 'chemoembolization' 27,591

#4. 'lenvatinib'/exp OR 'lenvatinib' 9,642

#3. #1 OR #2 231,770

#2. ('hepatocellular carcinoma'/exp OR 231,465 'hepatocellular carcinoma' OR (hepatocellular AND

('carcinoma'/exp OR carcinoma))) AND 'liver cell

carcinoma'

#1. 'liver cell carcinoma'/exp 231,758

**Cochrane Library databases:**

ID Search Hits

#1 MeSH descriptor: [Carcinoma, Hepatocellular] explode all trees 2805

#2 (hepatocellular carcinoma) OR (liver cancer):ti,ab,kw (Word variations have been searched) 18344

#3 #1 OR #2 18344

#4 MeSH descriptor: [Immune Checkpoint Inhibitors] explode all trees 326

#5 (immune checkpoint inhibitor) (Word variations have been searched) 2591

#6 #4 OR #5 2591

#7 MeSH descriptor: [] explode all trees 0

#8 (transarterial chemoembolization) OR (TACE):ti,ab,kw (Word variations have been searched) 1703

#9 #7 OR #8 1703

#10 MeSH descriptor: [] explode all trees 0

#11 (lenvatinib) (Word variations have been searched) 756

#12 #10 OR #11 756

#13 #9 AND #12 69

#14 #6 OR #13 2658

#15 #3 AND #14 341
