# Supplementary material for: The impact of immune checkpoint inhibitors on prognosis in unresectable hepatocellular carcinoma treated with TACE and lenvatinib: a meta-analysis
Source: Front Immunol. 2025 May 21;16:1573505. doi: 10.3389/fimmu.2025.1573505 (PMC12133757; doi:10.3389/fimmu.2025.1573505)
Supplement: Supplementary file 1 [file DataSheet1.zip › Supplementary files/Supplementary file 2 Quality evaluation of included studies.docx]

Supplementary file 2 Quality evaluation of included studies

| Study | Type of Study |  | Selection | |  |  | |  | Comparability | |  | Outcome |  |  |
| --- | --- | --- | --- | --- | --- | --- | --- | --- | --- | --- | --- | --- | --- | --- |
|  |  | 1 | 2 | 3 | | | 4 | | 5 | 6 | 7 | 8 | 9 | Score |
| Cai M, et al. 2022 | R | ☆ | ☆ | ☆ | | | ☆ | | ☆ |  | ☆ | ☆ |  | 7 |
| Chen S, et al. 2024 | R | ☆ | ☆ | ☆ | | | ☆ | | ☆ |  | ☆ | ☆ |  | 7 |
| Ding ZR, et al. 2024 | R | ☆ | ☆ | ☆ | | | ☆ | | ☆ |  | ☆ | ☆ |  | 7 |
| Guo P, et al. 2022 | R | ☆ | ☆ | ☆ | | | ☆ | | ☆ |  | ☆ |  |  | 6 |
| Jiang JY, et al. 2024 | R | ☆ | ☆ | ☆ | | | ☆ | | ☆ |  | ☆ | ☆ |  | 7 |
| Qu WF, et al. 2022 | R | ☆ | ☆ | ☆ | | | ☆ | | ☆ |  | ☆ |  |  | 6 |
| Sun B, et al. 2022 | R | ☆ | ☆ | ☆ | | | ☆ | | ☆ |  | ☆ | ☆ |  | 7 |
| Wang WJ, et al. 2023 | R | ☆ | ☆ | ☆ | | | ☆ | | ☆ |  | ☆ | ☆ |  | 7 |
| Wang YY, et al. 2023 | R | ☆ | ☆ | ☆ | | | ☆ | | ☆ |  | ☆ | ☆ |  | 7 |
| Wu HX, et al. 2023 | R | ☆ | ☆ | ☆ | | | ☆ | | ☆ |  | ☆ | ☆ |  | 7 |
| Xiang Z, et al. 2023 | R | ☆ | ☆ | ☆ | | | ☆ | | ☆ |  | ☆ | ☆ |  | 7 |
| Yang H, et al.2023 | R | ☆ | ☆ | ☆ | | | ☆ | | ☆ |  | ☆ | ☆ |  | 7 |
| Zhao S, et al. 2022 | R | ☆ | ☆ | ☆ | | | ☆ | | ☆ |  | ☆ | ☆ |  | 7 |
| Zhao YS, et al. 2024 | R | ☆ | ☆ | ☆ | | | ☆ | | ☆ |  | ☆ | ☆ |  | 7 |
| Zou X, et al. 2023 | R | ☆ | ☆ | ☆ | | | ☆ | | ☆ |  | ☆ | ☆ |  | 7 |

Note: R- Retrospective comparative study; RCT- Randomized Controlled Trial; - - Not Applicable; 1. Representativeness of exposed cohort; 2. Selection of non-exposed cohort; 3.Ascertainment of exposure; 4.Outcomeof interest was not present at start of study; 5. Study controls for age, sex, and marital status; 6. Study controls for any additional factors; 7.Assessment of outcomes; 8. Follow-up long enough for outcomes to occur; 9. Adequacy of follow-up.
