# Supplementary material for: The impact of immune checkpoint inhibitors on prognosis in unresectable hepatocellular carcinoma treated with TACE and lenvatinib: a meta-analysis
Source: Front Immunol. 2025 May 21;16:1573505. doi: 10.3389/fimmu.2025.1573505 (PMC12133757; doi:10.3389/fimmu.2025.1573505)
Supplement: Supplementary file 1 [file DataSheet1.zip › Supplementary files/Supplementary file 3.funnel plots.docx]

Supplementary file 3 funnel plots

Complete response

Partial response

Stable disease

Progressive disease

Objective response rate

Disease control rate

Overall survival

Disease free survival

Hypertension

Diarrhea

Hand-foot syndrome

Fatigue

Elevated AST

Elevated ALT

Decreased appetite

Hypothyroidism

Abdominal pain

Thrombocytopenia

Rash

Nausea
