# Supplementary material for: The impact of immune checkpoint inhibitors on prognosis in unresectable hepatocellular carcinoma treated with TACE and lenvatinib: a meta-analysis
Source: Front Immunol. 2025 May 21;16:1573505. doi: 10.3389/fimmu.2025.1573505 (PMC12133757; doi:10.3389/fimmu.2025.1573505)
Supplement: Supplementary file 1 [file DataSheet1.zip › Supplementary files/Supplementary file 4 Begg's and Egger's test for all included studies.docx]

Supplementary file 4 Begg's and Egger's test for all included studies

| Measured Outcomes | No. Studies | Begg's Test | | Egger's test |
| --- | --- | --- | --- | --- |
|  |  | Pr>\|z\|* | Pr > \|z\|** | P>\|t\| * |
| **Response** |  |  |  |  |
| Complete response | 15 | 0.139 | 0.155 | 0.63 |
| Partial response | 15 | 0.882 | 0.921 | 0.979 |
| Stable disease | 15 | 0.586 | 0.621 | 0.348 |
| Progressive disease | 15 | 0.882 | 0.921 | 0.351 |
| Objective response rate | 15 | 0.586 | 0.621 | 0.747 |
| Disease control rate | 15 | 0.051 | 0.052 | 0.061 |
| **Any Grade Adverse events** |  |  |  |  |
| Hypertension | 15 | 0.347 | 0.373 | 0.483 |
| Diarrhea | 12 | 0.17 | 0.193 | 0.089 |
| Hand-foot syndrome | 13 | 0.222 | 0.246 | 0.143 |
| Fatigue | 12 | 0.17 | 0.193 | 0.969 |
| Elevated AST | 6 | 0.015 | 0.024 | 0.075 |
| Elevated ALT | 7 | 0.453 | 0.548 | 0.321 |
| Decreased appetite | 9 | 0.835 | 0.917 | 0.961 |
| Hypothyroidism | 11 | 0.066 | 0.062 | 0.073 |
| Abdominal pain | 12 | 0.273 | 0.304 | 0.158 |
| Thrombocytopenia | 6 | 0.091 | 0.133 | 0.012 |
| Rash | 9 | 0.404 | 0.466 | 0.126 |
| Nausea | 13 | 0.807 | 0.855 | 0.478 |
| **Long-term outcome** |  |  |  |  |
| Overall survival | 11 | 0.128 | 0.152 | 0.244 |
| Disease free survival | 14 | 0.393 | 0.428 | 0.08 |

Note: * Values < 0.05 are considered statistically significant, which are in bold; ** Continuity corrected; NA- Not available.
